# Implementation of ICD-10 in Canada: how has it impacted coded hospital discharge data?

**DOI:** 10.1186/1472-6963-12-149

**Published:** 2012-06-10

**Authors:** Robin L Walker, Deirdre A Hennessy, Helen Johansen, Christie Sambell, Lisa Lix, Hude Quan

**Affiliations:** 1 Department of Community Health Sciences, University of Calgary, Calgary, Alberta, Canada; 2 Health Analysis Division, Statistics Canada, Ottawa, Ontario, Canada; 3 School of Public Health, University of Saskatchewan, Saskatoon, Saskatchewan, Canada; 4 Department of Community Health Sciences, University of Calgary, 3280 Hospital Dr. NW, Calgary, Alberta, Canada, T2N 4Z6

**Keywords:** International classification of disease version 10, Administrative data, Hospital records, Canada, Coding, Hospital discharge data

## Abstract

**Background:**

The purpose of this study was to assess whether or not the change in coding classification had an impact on diagnosis and comorbidity coding in hospital discharge data across Canadian provinces.

**Methods:**

This study examined eight years (fiscal years 1998 to 2005) of hospital records from the Hospital Person-Oriented Information database (HPOI) derived from the Canadian national Discharge Abstract Database. The average number of coded diagnoses per hospital visit was examined from 1998 to 2005 for provinces that switched from International Classifications of Disease 9^th^ version (ICD-9-CM) to ICD-10-CA during this period. The average numbers of type 2 and 3 diagnoses were also described. The prevalence of the Charlson comorbidities and distribution of the Charlson score one year before and one year after ICD-10 implementation for each of the 9 provinces was examined. The prevalence of at least one of the seventeen Charlson comorbidities one year before and one year after ICD-10 implementation were described by hospital characteristics (teaching/non-teaching, urban/rural, volume of patients).

**Results:**

Nine Canadian provinces switched from ICD-9-CM to ICD-I0-CA over a 6 year period starting in 2001. The average number of diagnoses coded per hospital visit for all code types over the study period was 2.58. After implementation of ICD-10-CA a decrease in the number of diagnoses coded was found in four provinces whereas the number of diagnoses coded in the other five provinces remained similar. The prevalence of at least one of the seventeen Charlson conditions remained relatively stable after ICD-10 was implemented, as did the distribution of the Charlson score. When stratified by hospital characteristics, the prevalence of at least one Charlson condition decreased after ICD-10-CA implementation, particularly for low volume hospitals.

**Conclusion:**

In conclusion, implementation of ICD-10-CA in Canadian provinces did not substantially change coding practices, but there was some coding variation in the average number of diagnoses per hospital visit across provinces.

## Background

In Canada and elsewhere, administrative hospital data are produced through review, abstraction and coding of data from in-patient charts after patients are discharged from hospital. The traditional roles of these data are to monitor health services utilization and to assess health services needs for administrative purposes. In the past two decades, nationally and internationally, administrative hospital data have been increasingly used by health services and population health researchers to study health care outcomes, effectiveness, appropriateness and utilization of health care services, and to investigate or monitor population health status and its determinants [1-5]. The widespread use of administrative hospital data has been facilitated by important advantages of the data, including their 1) readiness to be analyzed; 2) wide geographic coverage; 3) relatively complete capture of episodes of patient contact with the health system; and 4) relatively low cost to use. [6-8]. However, the use of these data for research purposes (i.e.purposes other than their primary use in funding and administration) is based on the assumption that they provide valid information about diagnoses, comorbidity and clinical services.

The World Health Organization’s (WHO) *International Classification of Diseases (ICD)* has become the international standard diagnostic classification for reporting mortality and most countries morbidity [9,10]. To date, substantial efforts have been made to validate the *ICD 9*^*th*^*Revision* (ICD-9) system used to code diagnoses and procedures recorded in hospital [11-16]. Many investigators have conducted validation studies focusing on comorbidities , clinical conditions, and complications of substandard care [15,17-21] and have found that administrative hospital data are accurately coded for severe or life-threatening conditions such as myocardial infarction and cancer, but that some conditions like rheumatologic disease are less accurately coded. The introduction of a new coding system, the *ICD 10*^*th*^*Revision* (ICD-10), by the WHO in 1992 has raised new questions about the coding accuracy and completeness of clinical information recorded in administrative data and whether there have been changes in the magnitude of coders’ errors between ICD-9 and ICD-10 coding systems. This is largely because ICD-10 codes uses a new alphanumeric system and each code in ICD-10 starts with a letter (i.e., A-Z), followed by two numeric digits, a decimal, and a digit (e.g., acute bronchiolitis due to respiratory syncytial virus is J21.0). In contrast, codes in ICD-9 begin with three digit numbers (i.e., 001–999), that are followed by a decimal and up to two digits (e.g., acute bronchiolitis due to respiratory syncytial virus is 466.11). Many ICD-10 codes are not directly convertible to corresponding ICD-9 codes. Many countries have found it necessary to develop their own ICD-10 clinical modifications to address country-specific needs. A modified version of the ICD-10, the ICD-10-CA, was approved for use in Canada in 1995 for hospital morbidity coding. This version contains more codes than previous versions, to help elaborate diagnoses and symptoms, as well as a new classification tool for interventions, *The Canadian Classification of Health Interventions* (CCI). Although the ICD-10-CA/CCI was approved relatively early for use in Canada, the timing of implementation of the new system varied greatly by province. In addition, the intensiveness of training of coders and the way in which the coded information was used (i.e. some provinces used diagnosis information to calculate funding requirements), also differed by province. The staggered introduction of the ICD-10-CA may have affected diagnoses and comorbidity data available in the administrative hospital data. Therefore the purpose of this study was to describe variation in diagnosis and comorbidity coding across the provinces and assess whether the change in coding classification has had an impact on Canadian hospital discharge data. Specifically, we investigated the average number of diagnostic codes and prevalence of clinically important comorbidities in hospital discharge data before and after ICD-10 implementation in Canadian provinces.

## Methods

### Study design

This was a descriptive study of diagnosis and comorbidity coding before and after implementation of ICD-10- CA coding in 9 Canadian provinces from 1998 to 2005. Quebec and the territories (Nunavut, Northwest Territories, Yukon) were excluded from the analyses due to lack of available data. The study was approved by the Conjoint Health Research Ethics Board at the University of Calgary.

### Data sources

This study examined 8 years (fiscal years 1998 to 2005) of hospital records from Statistics Canada’s Hospital Person-Oriented Information database (HPOI). The HPOI is a person-level dataset derived from discharge records of inpatients in most of the acute care hospitals and some psychiatric, chronic and rehabilitation hospitals across Canada [22]. The discharge records contain demographic (for example, date of birth, postal code), administrative (health number, admission and separation dates) and clinical information (up to 25 diagnoses and 10 procedures are listed for each hospital discharge) and are initially compiled into the Discharge Abstract Database (DAD) by the Canadian Institute for Health Information (CIHI). During processing at Statistics Canada, about 3% of DAD records for patients aged 12 or older were excluded because of missing or invalid health numbers [23]. CIHI collates the DAD from all the provinces and territories into a national dataset, which is continuously updated. The DAD is generated by medical coders and includes information on all patients admitted to hospital. Additionally, the DAD has a diagnosis type indicator, which permits the distinction between medical diagnoses that were present at the time of hospital admission. Thus the coders assign a one digit ‘diagnosis-type’ code to specify the timing of diagnosis (for example type 1 is pre-existing conditions that influence care or the hospital stay) [13]. In Canada, CIHI provides ICD coding guidelines [24] and an online coding query service (established in June 2001), however the implementation of coding rules and training of coders are the responsibility of each hospital or health region within each province/territory.

### Analysis

First, the average number of coded diagnoses per hospital visit was examined from 1998 to 2005 for 9 provinces (Newfoundland (NL), Prince Edward Island (PEI), Nova Scotia (NS), New Brunswick (NB), Ontario (ON), Manitoba (MB), Saskatchewan (SK), Alberta (AB), British Columbia (BC)) that switched from ICD-9 to ICD-10-CA during this period. In addition, the average number of type 2 diagnoses (i.e. diagnoses arising after hospital admission) and type 3 diagnoses (i.e. secondary diagnoses, present at hospital admission) were described. These types of diagnosis are commonly used by health services researchers to examine in-hospital complications, such as nosocomial infections (type 2) and to produce risk-adjusted outcomes (type 3).

Second, the distribution of the Charlson score and prevalence of the Charlson comorbidities [25] one year before and one year after ICD-10 implementation was examined (nationally, and by province). The Charlson index was initially developed to predict 1-year survival in medical patients admitted to a teaching hospital. This index is composed of 17 comorbidities, where each comorbidity is assigned a weighted score and then the weighted scores are summed to give an indicator of disease burden, the Charlson score. We used the ICD-10 and ICD-9 coding algorithms developed by Quan et al.[26] to derive the Charlson comorbidities and score for each discharge abstract (data not shown). In this multi-step process, ICD-10 coding algorithms were developed by translating the ICD-9-CM codes derived from Deyo’s method [27].

Finally, the proportion of records with at least one of the seventeen Charlson comorbidities one year before and one year after ICD-10 implementation were described by hospital characteristics. Important hospital characteristics included whether the facility was teaching or non-teaching (determined from the HPOI); whether the facility was located in an urban or rural setting (determined from the facilities’ postal codes available in the HPOI); and the volume of the facility, divided into quartiles (determined from the HPOI).

Statistical analyses were performed using SAS statistical software version 9.1 (SAS Institute Inc, Cary, North Carolina). Descriptive statistics were employed to report the mean number of diagnoses and Charlson comorbidities. We also assessed the median number of diagnoses but found it similar to the mean, thus have only reported the mean in study results.

## Results

### Change in coding systems

**Figure 1:**
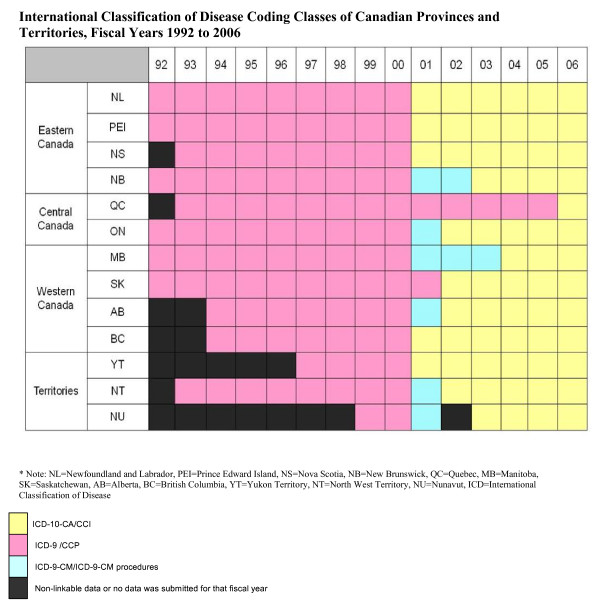
International Classification of Disease Coding Classes of Canadian Provinces and Territories, Fiscal Years 1992 to 2006.

Canadian provinces switched from ICD-9-CM to ICD-I0-CA over a six-year period. Implementation began in fiscal year 2001 for five provinces, with the last province, Quebec, switching in fiscal year 2006, see Figure 1. Provincial population, number of hospital/clinical units submitting data, and the number of hospital discharges before and after the switch to ICD-10-CA are described in Table 1. The number of hospital discharges remained relatively stable after ICD-10 implementation, with the largest increase seen in Alberta (ICD-9: 3,433 discharges per year, ICD-10: 3,736).

**Table 1:** Provincial characteristics one year before and one year after ICD-10 implementation

**Province**	**NL**		**PEI**		**NS**		**NB**		**ON**		**MB**		**SK**		**AB**		**BC**	
**Year before and after ICD 10 implementation**	2000	2001	2000	2001	2000	2001	2002	2003	2001	2002	2003	2004	2001	2002	2001	2002	2000	2001
**Population count (thousands)**	527.9	522.0	136.5	136.7	933.8	932.5	749.3	749.4	11,896.7	12,091.0	1,163.8	1,173.6	1,000.2	996.8	3,058.0	3,128.4	4,039.2	4,076.3
**Number of hospitals/ clinical units submitting data**	35	27	6	7	32	35	33	29	211	203	73	72	63	62	99	100	90	82
**Number of discharges/year**	1,251	1,205	900	952	1,912	2,000	1,671	1,761	11,257	11,183	2,737	2,798	2,904	3,027	3,433	3,736	3,892	3,975

* Data from Statistics Canada’s Hospital Person-Oriented Information database (HPOI)

Notes: *NL* Newfoundland and Labrador, *PEI* Prince Edward Island, *NS* Nova Scotia, *NB* New Brunswick, *ON* Ontario, *MB* Manitoba, *SK* Saskatchewan, *AB* Alberta, *BC* British Columbia, *ICD* International Classification of Disease

### Average number of diagnosis coded per hospital visit

**Figure 2:**
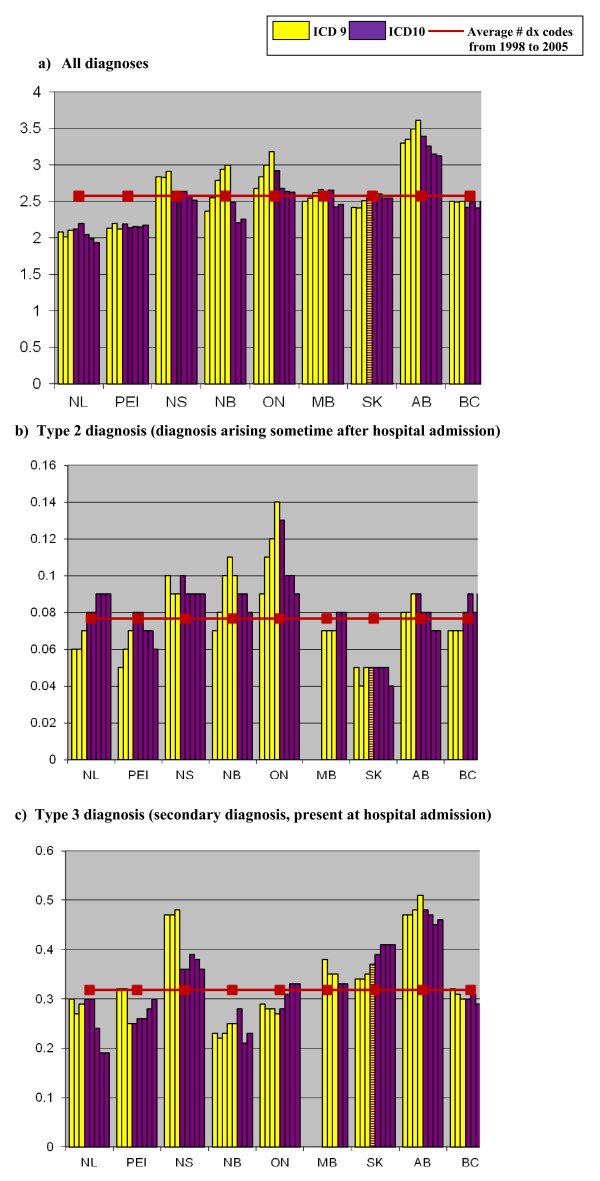
Average number of diagnosis codes per hospital visit by province and diagnosis type: fiscal years 1998 to 2005.

The average number of diagnoses coded per hospital visit for all code types over the study period was 2.58. Overall, AB coders coded the highest average number of diagnoses (3.33 diagnosis codes/hospital visit), while, the lowest number of diagnoses was coded in NL (2.06 diagnosis codes/hospital visit), see Figure 2a. After implementation of ICD-10-CA a decrease in the number of diagnoses coded was found in four provinces (NS, NB, ON and AB), whereas the number of diagnoses coded in the other five provinces (NL, PEI, MB, SK, BC) remained similar.

During the study period the average number of type 2 diagnosis (i.e. diagnoses arising sometime after hospital admission) coded per hospital visit was 0.08, with the highest number being coded in ON (0.11) and the lowest in SK (0.05), see Figure 2b. After the implementation of ICD-10-CA there was an increase in coding of type 3 diagnoses for MB, BC and NL, and a decrease in coding found in NB and ON.

From 1998 to 2005 the average number of type 3 diagnoses (i.e. secondary diagnoses, present at hospital admission) coded per hospital visit was 0.31, with the highest number being coded in AB (0.47) and the lowest in MB (0.22), see Figure 2c. A decrease in coding was found for PEI, NS, MB and AB. An increase in coding was found for ON and SK.

### Distribution of the Charlson score and prevalence of Charlson comorbidities before and after ICD-10 implementation

Across the spectrum of Charlson scores from 0 (indicating no burden of chronic disease) to 6+ (indicating very high burden of chronic disease) the distribution of scores did not change significantly from ICD-9 to ICD-10-CA, with the absolute differences ranging from 0.01% to 0.59%, see Table 2. The average Charlson score was also very similar after ICD-10 implementation, 0.64 (before) compared to 0.63 (after), Table 2. Additionally, the Charlson scores (grouped as 0, 1–2 and 3+ points) did not differ considerably across provinces, see Table 3.

**Table 2:** Distribution of the Charlson score one year before and after ICD-10 implementation

**Charlson score**	**ICD-9**	**ICD-10**	**Difference**
0	67.94	68.53	−0.59
1	15.41	14.90	0.51
2	8.98	9.00	−0.02
3	3.47	3.38	0.09
4	1.39	1.40	−0.01
5	1.89	1.96	−0.07
6+	0.92	0.83	0.11
Average Charlson score	0.64	0.63	0.01

**Table 3:** Distribution of the Charlson score one year before and after ICD-10 implementation, by province

**Province**	**Charlson score**	**ICD-9**	**ICD-10**	**Difference**
NL	0	71.01	69.09	1.92
	1-2	23.75	24.88	−1.13
	3+	5.24	6.03	−0.79
PEI	0	71.05	70.77	0.28
	1-2	24.31	24.13	0.18
	3+	4.64	5.10	−0.76
NS	0	64.93	67.06	−2.13
	1-2	26.84	25.72	1.12
	3+	8.23	7.22	1.01
NB	0	66.10	69.18	−3.08
	1-2	26.18	24.36	1.82
	3+	7.72	6.46	1.26
ON	0	66.42	67.46	−1.04
	1-2	25.14	24.38	0.76
	3+	8.44	8.16	0.28
MB	0	68.65	68.81	−0.16
	1-2	24.13	23.90	0.23
	3+	7.22	7.29	−0.07
SK	0	71.45	70.24	1.12
	1-2	23.32	23.64	−0.32
	3+	5.23	6.12	−0.89
AB	0	68.31	68.88	−0.57
	1-2	23.53	22.89	0.64
	3+	8.16	8.23	−0.07
BC	0	71.00	70.53	0.47
	1-2	22.49	22.79	−0.30
	3+	6.51	6.68	−0.17
Canada	0	67.95	68.49	−0.54
	1-2	24.39	23.91	0.48
	3+	7.67	7.60	0.07

Similarly, the prevalence of at least one of the seventeen Charlson conditions was relatively stable after ICD-10 implementation across 9 Canadian provinces, see Table 4. The absolute difference in prevalence of at least one of the seventeen Charlson conditions between ICD-9 and ICD-10-CA ranged from 0.1% to 4.1%. More specifically, NL showed almost no change in prevalence (0.1%) compared to NB (4.1%).

**Table 4:** Prevalence of at least 1 of the 17 Charlson comorbidities in one year before and after ICD-10 implementation, by province

**Province**	**ICD-9**	**ICD-10**	**Difference**
NL	29.0	28.9	0.1
PEI	28.9	29.2	−0.3
NS	35.1	32.9	2.2
NB	34.9	30.8	4.1
ON	33.6	32.5	1.1
MB	31.3	31.6	−0.3
SK	28.5	29.8	−1.3
AB	31.7	31.1	0.6
BC	29.0	29.5	−0.5

Notes: *NL* Newfoundland and Labrador, *PEI* Prince Edward Island, *NS* Nova Scotia, *NB* New Brunswick, *ON* Ontario, *MB* Manitoba, *SK* Saskatchewan, *AB* Alberta, *BC* British Columbia

When stratified by hospital characteristics, the prevalence of at least one Charlson condition decreased after ICD-10-CA implementation, see Table 5. This was particularly noticeable for low-volume hospitals.

**Table 5:** Prevalence of at least 1 of 17 Charlson comorbidities in one year before and after ICD-10 implementation, by hospital characteristics

**Hospital Characteristic**	**ICD-9**	**ICD-10**	**Difference**
*Teaching Status*			
Teaching	36.6	32.1	4.5
Non-Teaching	34.9	29.2	5.7
Location			
Urban	35.5	29.9	4.6
Rural	34.8	30.1	3.7
*Patient Volume*			
High (>75%)	35.4	37.5	−2.1
Medium (50-75%)	33.9	27.4	6.5
Low (25-50%)	36.5	27.3	9.2
Very Low (<25%)	37.7	29.6	8.1

Notes: *ICD* International classification of disease

## Discussion

This study investigated whether implementation of the ICD-10-CA diagnostic coding system has affected coded administrative hospital data in Canada. First, we assessed whether the number and type of diagnoses coded was affected by the changeover. Second, we investigated whether the coding of the Charlson score and comorbidities changed from the year before to the year after ICD-10-CA implementation. Overall, our results suggest that there is variation across provinces in the average number of diagnosis codes per hospital visit, both before and after the implementation of ICD-10-CA. Additionally, when ICD-10-CA was implemented, variation in coding between provinces was also found in type 2 and 3 diagnoses. The impact of the implementation of ICD-10-CA was minimal when examining the distribution of the Charlson score and the prevalence of the Charlson comorbidities.

A potential reason for the overall minimal changes seen in coding after ICD-10 was implemented is coder training. In Canada, coder training is regulated at a provincial level and changes from year to year, region to region, and hospital to hospital (documentation of coder training is limited). Exceptionally, when ICD-10 was implemented CIHI delivered a two-day workshop that was given to all provinces/territories when they implemented ICD-10-CA to ensure a smooth transition. Furthermore, as ICD-10 was implemented in Canada over a 6-year period CIHI had time to discover specific issues coders were having and change the training accordingly. Our study findings are also consistent with a previous study conducted by Quan et al. [28] who assessed the validity of the ICD-10 Canadian hospital discharge data to determine whether there were improvements in the validity of coding for clinical conditions compared with ICD-9. The study found that the data quality was stable between the two coding systems, although the validity differed between coding versions for some clinical conditions. A recent study in Switzerland [29] evaluated the accuracy of comorbidity coding overtime after the introduction of ICD-10 and in fact found slight improvements in coding. In Canada, assessing coding changes overtime after the implementation of ICD-10 is needed (see future research below).

Although our overall results reflect minimal changes in coding between the two systems we did observe that four provinces (NS, NB, ON and AB) had a decrease in the average number of all diagnoses coded the year after implementation of ICD-10. A potential explanation for this decrease is related to the fact that health record coders were learning a new coding system. Although provinces/territories received ICD-10 training by CIHI it was expected that after implementation of ICD-10-CA a learning curve would be present as health record technicians were tasked with becoming familiar with new classifications, new software, and a new discharge abstract. Therefore they were likely to have to spend more time coding appropriate diagnoses. For example, many coders would reference the ICD-9 manual to find corresponding ICD-10 codes which would take up a significant amount of time allotted to the chart abstraction resulting in fewer conditions being coded. However, we observed that the number of diagnoses coded in these four provinces did not subsequently increase after the initial drop in coding when ICD-10-CA was implemented. This may indicate that the coding practice guidelines may have changed after implementation of ICD-10-CA. In AB, for example, due to the large number of secondary diagnoses and the limited time available to code each patient chart (30 minutes per chart), coders were instructed to focus on common conditions (such as diabetes, hypertension, heart disease, etc.) when ICD-10-CA was implemented. Therefore, in AB, minor conditions were less likely to be coded, resulting in a decrease in the average number of diagnosis codes per hospital visit regardless of the type of diagnosis. In Canada, regular auditing of coding is done at a national level by the CIHI. However the audit is periodical and may not have occurred in the year after ICD-10 was implemented in the 4 provinces that had a decrease in coding.

Before ICD-10-CA implementation the four provinces that saw decreases in number of diagnoses coded had higher average number of diagnoses coded compared with the five provinces in which there was no obvious change in coding for all diagnoses (NL, PEI, MB, SK, BC). However, after implementation of ICD-10-CA the average number of all diagnoses for these four provinces began to decline towards the overall average number of diagnoses for the other five provinces. A potential explanation for the coding patterns may be related to the provincial variation in health care funding. Canadian provinces currently receive annual lump sums from the government to cover hospital operational expenses, a payment model known as block funding. However the method by which these resources are allocated to hospitals, within provinces, varies and is unfortunately very difficult to track. While some provincial hospitals use case-mix grouping methodology for determining hospital funding, which takes diagnostic codes into account [30,31], others provinces allocate funding based on the age and sex breakdown of the patient population only. Therefore, depending on the type of payment system, some provinces may code more diagnoses than others [31].

Because coded hospital data are also commonly used for outcomes research, we assessed the distribution and prevalence of the Charlson score and comorbidies (a common risk adjustment tool) before and after the implementation of ICD-10-CA. This study found that there were very minor changes in the distribution and prevalence of Charlson score and comorbidities between ICD-9-CM and ICD-10-CA. This finding is consistent with the previous study conducted by Quan et al. [28] which shows that data quality was stable between two coding systems. Therefore, ICD-10-CA implementation most likely did not have a significant impact on the coding of important comorbid conditions that are commonly used in risk adjustment.

This study has some limitations. We only described the coding practices among nine provinces and were unable to assess the remaining province and territories due to data availability issues. We were unable to describe in detail patient volume levels (Table 5) which would have been good to have, but not possible due to data approval issues. Additionally, this study was unable to quantify the impact of ICD-10-CA implementation on other data quality elements, like accuracy and consistency. Neither did this study attempt to assess the impact of ICD-10-CA implementation on overall patient complexity, because this concept is ultimately related the average number of diagnoses per hospital visit.

Future research includes a Canadian study assessing accuracy and consistency of ICD-10 coding. Since the introduction of ICD-10 coding systems, studies have assessed accuracy of coding [19,25,29,32]. For example, an Australian study [19] demonstrated that the validity of ICD-10 administrative data was high after two years of implementation of the new system. Another study compared the accuracy of ICD-10 coding in hospital administrative data versus medical charts [32]. This study found that a substantial percentage (29%) of records had inaccurate diagnostic codes and concluded that a routine data coding audit would be useful to improve the accuracy of routine diagnostic codes.

Quan et al [28] assessed teaching hospitals 6 months after implementation of ICD-10 in Canada, however this study did not assess long term impact of ICD-10. Thus, in the future we need a further study to assess the affect of change over to ICD-10 by region, type of hospital and over time. Studies have evaluated ICD-9 and ICD-10 coding for hypertension and diabetes and found no difference in coding between the 2 systems [33,34], however multiple conditions, including less prevalent conditions, need to be assessed.

## Conclusions

In conclusion, implementation of ICD-10-CA in Canadian provinces did not substantially change coding practices for common conditions. There was some coding variation in the average number of diagnoses per hospital visit across provinces. This information should be considered by researchers and policymakers when comparing trends in hospitalization for particular diseases across time. In the future, more countries will be implementing ICD-10, including the United States who plan to introduce ICD-10 in 2013 [35]. Currently the World Health Organization is in development and production of ICD-11, which is planned to be released in 2015. Therefore the impact of coding system changes and their validation will become increasingly important, both nationally and internationally. Future Canadian research will assess ICD-10 coding accuracy and consistency.

## Abbreviations

ICD, International classifications of disease; CCI, Canadian classification of health interventions; DAD, Discharge abstract database; CIHI, Canadian institute of health information; NL, Newfoundland; PEI, Prince Edward Island; NS, Nova Scotia; NB, New Brunswick; ON, Ontario; MB, Manitoba; SK, Saskatchewan; AB, Alberta; BC, British Columbia.

## Competing interests

The authors declare that they have no competing interests.

## Authors’ contributions

RW contributed conception and design, drafted the manuscript, prepared tables, and interpreted data. DH contributed to the conception and design, helped draft and critically revised the manuscript. HJ participated in study design, provided the data, and critically revised the manuscript. CS contributed to conception and design and performed statistical analyses. LL participated in the interpretation of data and critically revised the manuscript for intellectual content. HQ contributed to the conception and design, oversaw the project including analysis and writing, interpreted data and critically revised the manuscript for intellectual content. All authors have read and approved the final manuscript.

## Pre-publication history

The pre-publication history for this paper can be accessed here:

http://www.biomedcentral.com/1472-6963/12/149/prepub
